# Twisting tongues to test for conflict-monitoring in speech production

**DOI:** 10.3389/fnhum.2014.00206

**Published:** 2014-04-16

**Authors:** Daniel J. Acheson, Peter Hagoort

**Affiliations:** ^1^Neurobiology of Language Department, Max Planck Institute for PsycholinguisticsNijmegen, Netherlands; ^2^Donders Institute for Brain, Cognition and Behaviour, Radboud UniversityNijmegen, Netherlands

**Keywords:** speech production, error-related negativity (ERN), monitoring, flanker task, ERP/EEG, cognitive control, tongue twister, N2

## Abstract

A number of recent studies have hypothesized that monitoring in speech production may occur via domain-general mechanisms responsible for the detection of response conflict. Outside of language, two ERP components have consistently been elicited in conflict-inducing tasks (e.g., the flanker task): the stimulus-locked N2 on correct trials, and the response-locked error-related negativity (ERN). The present investigation used these electrophysiological markers to test whether a common response conflict monitor is responsible for monitoring in speech and non-speech tasks. Electroencephalography (EEG) was recorded while participants performed a tongue twister (TT) task and a manual version of the flanker task. In the TT task, people rapidly read sequences of four nonwords arranged in TT and non-TT patterns three times. In the flanker task, people responded with a left/right button press to a center-facing arrow, and conflict was manipulated by the congruency of the flanking arrows. Behavioral results showed typical effects of both tasks, with increased error rates and slower speech onset times for TT relative to non-TT trials and for incongruent relative to congruent flanker trials. In the flanker task, stimulus-locked EEG analyses replicated previous results, with a larger N2 for incongruent relative to congruent trials, and a response-locked ERN. In the TT task, stimulus-locked analyses revealed broad, frontally-distributed differences beginning around 50 ms and lasting until just before speech initiation, with TT trials more negative than non-TT trials; response-locked analyses revealed an ERN. Correlation across these measures showed some correlations within a task, but little evidence of systematic cross-task correlation. Although the present results do not speak against conflict signals from the production system serving as cues to self-monitoring, they are not consistent with signatures of response conflict being mediated by a single, domain-general conflict monitor.

## INTRODUCTION

Producing speech is one of the most common actions in which we engage. Like any other action, fluent and correct speaking requires self-monitoring. For many years, the dominant theory of monitoring in speech production has been the Perceptual Loop Theory (Levelt, 1983), in which signals from different stages of production planning are sent through the comprehension system and compared to an intended message. Recent research and modeling has suggested, however, that conflict signals arising within the production itself might serve as a critical cue to self-monitoring (Nozari et al., 2011). Such signals have been well-documented outside of language, with one prominent framework suggesting that all action domains might be monitored via a domain-general mechanism sensitive to detecting response conflict (e.g., Botvinick et al., 2001). This latter framework, however, has almost exclusively been investigated using manual responding in non-linguistic tasks. The goal of the present study is to test the viability of monitoring for response conflict in production by assessing whether similar electrophysiological and behavioral signatures of conflict are present in a speech production task and a standard, non-linguistic measure of conflict, the flanker task.

The Perceptual Loop account of monitoring in speech production provides a relatively simple and elegant framework as it does not require any additional machinery beyond that which is already provided by the comprehension system. According to this account, individuals monitor themselves after three stages of the speech production process: message retrieval, phonological encoding, and articulation. The Perceptual Loop account has recently been criticized, however, both on the grounds that internal self-monitoring does not seem to engage the speech comprehension (Huettig and Hartsuiker, 2010), and because of patterns of dissociations in patients who show impairments in comprehension but intact speech monitoring (see Nozari et al., 2011). Such criticisms have led numerous researchers to suggest that there may be specific monitors for different stages of production planning (see Postma, 2000 for a review).

An alternative to individual monitors at different stages of production planning is a more general account put forward by Nozari et al. (2011) who hypothesized that the production system might be monitored via domain-general, action monitoring systems that are sensitive to response conflict. Such conflict occurs when two or more representations are highly active prior to responding. In the case of language production planning, such conflict might arise, for instance, when two words with a similar meaning are both active at the same time (e.g., couch, sofa), or two speech sounds are concurrently active (e.g., */p/* and /b/). Using the two-step model of speech production of Dell and O’Seaghdha (1991), Nozari et al. (2011) were able to demonstrate that signals generated as the difference between the two most active elements at semantic and phonological levels of representation were good predictors of whether the model generated a lexical or phonological error. Moreover, the magnitude of these signals served as a good predictor of the speech production performance of a number of aphasic patients and the likelihood that they would detect an error. The authors tied this notion of conflict to a more overarching, action-monitoring framework provided by the response conflict model (Botvinick et al., 2001; Yeung et al., 2004).

According to the response conflict model, a region of the medial prefrontal cortex, the dorsal anterior cingulate cortex (ACC) is sensitive to situations where two competing responses are both active at the same time. This response conflict signal serves as a cue to regions of the dorsal lateral prefrontal cortex (DLPFC), which maintains task-relevant information and exerts control by biasing signals to task-specific brain regions to resolve the conflict. The response conflict model is based upon a relatively large cognitive control literature indicating ACC involvement in choice response tasks with manipulations of stimulus-response congruency such as the Flanker (Eriksen and Eriksen, 1974), Simon (Simon, 1969), and Stroop (Stroop, 1935) tasks. In these tasks, response conflict is induced either due to the incongruency between an intended response and the surrounding material (Flanker), a spatial position (Simon), or a pre-potent response (Stroop). Although there are a number of competing hypotheses about what the ACC is doing in such tasks (Holroyd and Coles, 2002; Rushworth et al., 2004; Aarts et al., 2008; Alexander and Brown, 2011), stable and replicable electrophysiological signatures of errors of commission and stimulus-response congruency have been established.

One such electrophysiological signature is the ERN, a centrally-distributed negativity that appears between 0 and 100 ms after an error has been committed (Falkenstein et al., 1991; Gehring et al., 1993). The ERN has been source-localized to the ACC in electroencephalography (EEG; Dehaene et al., 1994), and appears to have the same source regardless of output modality (e.g., hand vs. foot; Holroyd et al., 1998). Furthermore, the ERN is relatively stable within an individual across choice reaction time tasks (Riesel et al., 2013). As such, the ERN has been argued to be a marker of a domain-general, performance monitoring system, although researchers vary as to whether ERN marks response conflict (Yeung et al., 2004), reinforcement learning (Holroyd and Coles, 2002), the likelihood that an error will be committed (Brown and Braver, 2005), or the violation of an expected action outcome (Alexander and Brown, 2011).

One argument in favor of the response conflict account (cf. Alexander and Brown, 2011) is that it is highly correlated with the N2, a stimulus-locked negativity that is greater for incongruent relative to congruent trials (see Yeung et al., 2004). The N2 corresponds to the second negative peak in the ERP signal, and occurs within 200–400 ms following a stimulus. It has the same scalp distribution as the ERN, and occurs across a wide range of conflict-inducing tasks (for a review, see Folstein and van Petten, 2008). Furthermore, the N2 is sensitive to the amount of conflict across a block of trials, with the magnitude decreasing as the ratio of incongruent to congruent trials increases (Gratton et al., 1992), and the latency correlated with reaction times (Nieuwenhuis et al., 2003). These sets of results indicate that both the ERN and N2 are consistent markers of incongruency across stimulus-response dimensions and errors of commission, although to date they have been studied almost exclusively outside of language production.

Both markers, however, have been documented in language tasks with varying needs for production. The first demonstration of an ERN in language production came from Masaki et al. (2001) who showed an ERN for incorrect trials in the Stroop task with vocal responding. A series of studies by Ganushchak and Schiller (2006) also demonstrated that an ERN is present for errors in a phoneme-monitoring task while people covertly named pictures, and is also larger for semantically-related compared to unrelated blocks in a blocked-naming paradigm (Ganushchak and Schiller, 2008). This latter result was taken to indicate that the ERN can be sensitive to conflict arising not only from the response-level, but also from more abstract levels of representation (i.e., semantics). Whether one should expect similar brain signatures to representational vs. response conflict has been major issue of study in the cognitive control literature (see Milham et al., 2001; Egner, 2008), and we return to this point in the general discussion.

Recent research has also indicated that ERN-like components are also present during correct production as well. Using a picture naming paradigm, Riés et al. (2011) not only demonstrated an ERN on incorrectly named pictures, but also showed a relatively smaller negativity occurring in the same time window for correct responses. This negativity was argued to be a marker of an error-monitor system that is active both on correct and incorrect trials. A recent study using bilingual picture naming also demonstrated that the magnitude of an ERN-like component is sensitive to response conflict (Acheson et al., 2012). Acheson et al. (2012) showed that a relatively late (100–200 ms) frontal–central negativity was larger when bilingual German/Dutch speakers named pictures that were cognates (e.g., *Haus/huis* – English: house) relative to those that are not (*Frosch/kikker*; English – frog). The authors argued that despite having shared semantics and phonology which can lead to faster speech initiation times (i.e., the cognate facilitation effect; Costa et al., 2000), cognates might also induce response conflict as they differ during phonetic encoding and articulation – the response-levels of speech production. The timecourse of this response-locked negativity was somewhat later than is typically observed for the ERN, which may have occurred either because the conflict itself did not occur until later in the word (all began with the same speech sounds), or alternatively, because the negativity reflected something akin to the feedback-related negativity (Holroyd and Coles, 2002).

The study by Acheson et al. (2012) is significant in demonstrating that speakers are sensitive to response conflict during speech production, and that this can occur even after responding has been initiated. In addition to this response-locked signature, stimulus-locked indicators of conflict have also been observed using the SLIPs paradigm (Baars et al., 1975). Using overt-production, Moeller et al. (2007) demonstrated that 400–600 ms after stimulus-onset, a frontal–central negativity was observed that was larger before a speech error occurred relative to when it did not, and this negativity was source localized to an area just superior to the ACC, the pre-supplementary motor area (pre-SMA). More recent research using the same SLIPs paradigm demonstrated that this negativity is larger when the experimentally-induced error is about to produce a taboo word (Severens et al., 2011). Both of these studies were taken to indicate that the system responsible for monitoring speech is sensitive to conflicting responses prior to speaking.

The stimulus-locked N2 has also been found in numerous ERP studies of language. For instance, many studies have combined covert production with a Go/No-Go task requiring button presses. Such studies have shown that the timing of the N2 difference between Go/No-Go trials is sensitive to the timecourse of production planning, with the N2 for semantic decisions preceding phonological ones (e.g., Schmitt et al., 2000; Rodriguez-Fornells et al., 2002). Numerous studies using bilingual language paradigms have also found evidence of N2 modulation (see Rodriguez-Fornells et al., 2006 for a review). For example, the Go/No-Go paradigm has been used to demonstrate cross language activation at the phonological level (e.g., Rodriguez-Fornells et al., 2005), and an N2 has been observed in language switching paradigms requiring overt production (e.g., Christoffels et al., 2007; Verhoef et al., 2009).

The picture-word interference paradigm has also provided evidence of ACC involvement in language production. For instance, the N2 has been implicated in the picture-word interference paradigm for the need to inhibit non-linguistic distractors (e.g., “XXXX”; Hirschfeld et al., 2008), although later components seem to show sensitivity to linguistic distractors. For instance, phonologically-related distractors produce a larger frontal–central negativity than unrelated distractors starting around 400 ms after stimulus onset (Dell’Acqua et al., 2010). FMRI research has also revealed that portions of the ACC are sensitive to phonological relative to unrelated distractor words in the picture-word interference paradigm (de Zubicaray et al., 2001), although it should be noted that the region of the ACC observed in this study was more anterior and inferior to that which has been observed in the studies of using choice response tasks. What is noteworthy about the studies indicating that the N2 is sensitive to linguistic manipulations is that they all involve tasks in which participants must inhibit a response, thus it is unclear whether these effects reflect conflict monitoring or inhibition.

The research reviewed above thus highlights a number of similarities between mechanisms and signatures of response conflict monitoring outside of language and those that might occur within the production system itself. Although the brain-region responsible for monitoring conflict – the ACC – is purported to be domain-general, to date, no direct comparison has been made to test for comparable ACC engagement in language production and conflict-inducing, choice-response tasks. The present research was designed to address whether similar signatures of response conflict are present across these domains, and furthermore, whether these signatures are related to each other.

In order to induce response conflict in language production, native speakers of Dutch rapidly read phonotactically legal nonwords organized in tongue twister (TT) and non-TT patterns (see Wilshire, 1999 and Methods section below).The choice of nonwords in the present study was designed to focus on the phonological encoding and articulation-levels of production planning while limiting influences from lexical and semantic representations. The TT pattern causes more errors than non-TT, and was thus taken to be our high-conflict condition. Based on the above-described research, a number of predictions can be made if the same mechanisms of conflict monitoring are present in speech and non-speech tasks. First, errors should elicit an ERN in the response-locked analysis in both tasks, and their scalp topography should be the same. Second, differences between high and low conflict conditions should be observed in the N2 time window, with high conflict conditions more negative than low conflict conditions (i.e., TT > non-TT; flanker incongruent > flanker congruent). Third, there should be correlations between people’s behavioral performance and EEG signatures of conflict within both tasks. Finally, if the process of response conflict monitoring and error detection is domain-general, then there should be correlations between EEG signatures and people’s behavioral performance across both tasks.

## METHODS

### PARTICIPANTS

Forty eight native Dutch speaking participants (37 female) were recruited from the MPI subject database and were paid €8/h for their participation. Participants ranged between 17 and 26 years of age (M = 21.1, SD = 2.4), and had normal or corrected-to-normal vision. None had reading or speaking problems as indicated by a self-report questionnaire. Data from three participants was excluded due to recording errors during data collection. Data from one additional participant was excluded from the flanker analysis due to poor EEG signal, and four from the TT task due to poor EEG signal (two participants) voice key errors (two participants; see below). This left a total of 44 subjects for analysis in the flanker task, and 40 for analysis in the TT task.

### PROCEDURE

Participants began each session with an informed consent that followed the guidelines of the Declaration of Helsinki that was approved by a local ethics board.

#### Behavioral Tasks

***Flanker task.*** In the flanker task, participants were instructed to respond with a left or right button press according to the direction of a middle arrow surrounded by other arrows (see **Table 1**). In half of the trials flanking arrows pointed in the same direction as the middle arrow (i.e., congruent trials), and in the other half they pointed in the opposite direction (incongruent trials). Half of the trials required a left button press and half a right button press.

**Table 1:** Example stimuli for flanker and tongue twister tasks.

	High conflict	Low conflict
	Incongruent	Congruent
Flanker	>><>>	>>>>>
	**Tongue twister**	**Non-tongue twister**
Tongue twister	k*ag* y*eef* y*ag* k*eef*	k*ag* k*eef* y*ag* yeef
Onset	A B B A	A A B B
Rhyme	*C D C D*	*C D C D*

Each trial began with a fixation cross presented for 500 ms followed by the flanker stimulus for 100 ms. Following the stimulus, a blank screen was presented until the subject pressed a button, up to a maximum of 2500 ms. Following the response, a blank screen was presented for 500 ms followed by an inter-trial interval which varied randomly between 1000 and 1200 ms in which participants were encouraged to blink.

Participants were seated approximately 70 cm away from a computer monitor, and stimuli were presented in the middle of the screen. Participants were presented with a total of 480 trials, the first 60 of which constituted a practice block. Each block was divided into 60 randomly ordered trials, and the number of left/right responses as well as congruent/incongruent trials was balanced within a block. After each block, participants received feedback for their performance. In order to elicit errors, participants were encouraged to respond as quickly as possible. During feedback, participants were encouraged to respond more quickly if their average reaction time was slower than 550 ms or if their accuracy was above 90%.

***Tongue twister task.*** In the TT task, participants were required to repeatedly and rapidly read sequences of four legal nonword stimuli (i.e., pseudowords). Single-syllable nonwords were constructed to abide by Dutch phonotactics, and were composed of CVC, CVCC, CCVC, or CCVCC consonants and vowels. 60 TT patterns were constructed by using an alternating rhyme (i.e., CDCD) with syllable onsets ordered in either a TT (ABBA) or non-TT (AABB) pattern (see **Table 1**). The use of nonwords in this experiment was specifically motivated by the fact that we wanted to isolate difficulty during phonological encoding and articulation in the absence of lexical-semantic influences.

Trials began with participants familiarizing themselves with the nonwords prior to rapid reading. Each nonword was presented individually, and participants were instructed to read each nonword out loud. Each nonword was preceded by a 250 ms fixation, and was displayed for 1250 ms. After being familiarized with the four nonwords, a blank screen was presented for 2000 ms. Halfway through this presentation participants heard a 600 ms tone, which served as a cue that the rapid reading of the nonwords was about to begin.

During rapid reading, each nonword was then presented in the middle of screen for 500 ms followed by a 150 ms fixation cross. Participants were instructed to read each nonword out loud as it was presented on the screen. This procedure was repeated for each nonword, and the pattern of four nonword was repeated three times. Thus, participants spoke each sequence of four nonwords aloud three times, resulting in a production of 12 nonwords during the rapid reading portion of the trial.

Following repetition of the last nonword, participants were presented with a screen asking them whether they had made an error, and responses were recorded with a yes/no button press. This portion of the experiment was subject-paced, and afforded them the opportunity to take a break if desired. Following participant responses about whether they made an error, a 4000 ms inter-trial interval began during which participants were instructed to blink if necessary.

Participants began with six practice trials followed by 60 experimental trials broken up into blocks of 20 trials. The total number of TT and non-TT was balanced within a block, and each pattern was presented once. Participants were encouraged to take a break every 20 trials, during which time any electrodes that were showing poor signal could be adjusted.

#### EEG Recording

Electroencephalography was recorded in an electromagnetically-shielded room with 60 active surface electrodes placed in an equidistant montage (Acticap, Brain Products, Herrsching, Germany). An electrode on the left mastoid served as a reference with a forehead electrode as the ground. Vertical and horizontal eye-movements were recorded using electrodes on the cap in addition to one electrode placed below the left eye. EEG data were digitized at a rate of 500 Hz, and impedances were kept below 20 KΩ during the experiment.

### DATA PROCESSING AND ANALYSIS

#### Behavioral

***Flanker task.*** Prior to data analysis, all trials with responses <200 ms were excluded. The analysis of reaction times only included correct trials.

***Tongue twister task.*** Participant responses were transcribed and coded for whether there was an error or not. Three types of errors were coded: omissions (i.e., not responding), item ordering errors (i.e., saying one of the items in the list at the wrong time) and phoneme ordering errors (i.e., saying a phoneme in the wrong place). Note that errors coded as item ordering errors (e.g., saying the target *tiff deev diff teev* as *tiff teev diff* deev) could also correspond to a phoneme ordering error. Phoneme ordering errors thus only included errors that did not result in production of one of the items in the list. Omission errors were very rare (<0.3% of response) and were included in the overall error analysis for tongue-twister effects, but were not included in the EEG analysis as there was no vocal onset for these types of errors. Participants often spontaneously self-repaired their utterance (e.g., “*deh-tiff”*), thus, in addition to coding for errors, we also coded for whether a trial contained a self-correction. Such self-correction is an indication of people monitoring and correcting their performance.

***Statistical analysis.*** All behavioral comparisons between conditions were made with dependent-samples *t*-tests.

#### EEG

***Preprocessing.*** Preprocessing of the data was performed with Brain Vision Analyzer version 2.0 (Brain Products). Practice trials were removed, and the EEG data was re-referenced to the average of both mastoids. Horizontal and vertical eye-channels were calculated, and eye-blinks and horizontal movements were isolated using independence components analysis (ICA). Visual inspection of these components resulted in the average removal of 2.3 components (SD = 0.66), thus, on average, one blink and one eye-movement component was removed per subject.

Following removal of artifacts with ICA, data was bandpass filtered between 0.05 and 15 Hz in order to remove most of the motor artifact that may have been present. Although we would have preferred to utilize the BSS-CCA technique espoused by Riés et al. (2011) for data cleaning, after using this technique with the current dataset, significant motor artifact remained in the signal. Thus, we opted for a more traditional bandpass filter. Channels that were identified as noisy during recording were imputed for eight subjects using spherical spline interpolation (Mean = 1.3 channels, SD = 0.5). Data was epoched (see below), and any trial with a reaction time faster than 250 ms was excluded. Non-ocular artifacts were removed based on trials with amplitude above or below ±200 μV, or with a voltage step of 100 μV within 200 ms.

In the TT task, this data cleaning procedure resulted in the exclusion of 37% of the trials (SD = 18%) on average. Four subjects were excluded after this cleaning due to a low number of trials remaining. Two because of oversensitivity in the voice key, and two due to poor EEG signal overall. This left 40 subjects for further analysis, and with an average of 210 trials per TT condition, and a minimum of 82.

In the flanker task, the data cleaning procedure resulted in the exclusion of 13% of trials (SD = 1%) on average. No subjects were excluded due to poor signal or RTs that were too fast. This left an average of 183 trials on average per flanker condition, with a minimum of 132 trials.

***Stimulus-locked epochs.*** Epochs were extracted according to the onset of either the flanker stimulus or the nonword. For both tasks, an epoch length of 800 ms was chosen, from -200 ms before to 600 ms after the onset of the stimulus. A baseline period of -100 to 0 ms was used for both tasks. Only correct responses were used for the stimulus-locked analysis. For the TT task, this excluded any trial coded as an error or including disfluency.

***Response-locked epochs.*** Epochs of 700 ms were extracted relative to the onset of button presses in the flanker task and triggering of the voice key in the TT task (-300 ms before to 400 ms after). In line with previous studies of the ERN, trials were baseline corrected from -300 to -100 ms before the response, and analyses only included subjects who had six or more errors after the data cleaning procedures (see Olvet and Hajcak, 2009). It is important to note that errors were more likely in TT trials, and that the overall ERP for these trials was more negative than for correct trials prior to responding (see below). As such, use of this pre-response baseline may have differentially shifted correct and incorrect trials in the TT task. After excluding trials based on the rejection criterion described above, 30 subjects were left for the error analysis in the TT task with an average of 16 errors each (SD = 7.35). No subjects were excluded from the error analysis of flanker task, and subjects had, on average, 27 errors each (SD = 16.7).

***Statistical tests.*** Given a priori predictions about the N2 and ERN in the flanker task and the ERN in the TT task, statistical analyses focused on time windows surrounding these effects (N2: 250–350 ms post-stimulus; ERN: 0–100 ms post-response). Given the large difference in the number of correct and erroneous trials, there was a risk of bias in estimates of peak-to-peak amplitude, hence, we analyzed mean amplitude differences within the time windows of interest (see Luck, 2005). Given the typical distributions of the ERN and N2 effects, statistical analyses focused on five central electrodes going from frontal to posterior: FC, FCz, Cz, PCz, Pz. Data were submitted to a 5 (Channel Position) × 2 (Trial Type) repeated measures ANOVA, with Trial Type defined as error and correct trials for the response-locked analysis, and congruent/incongruent for the stimulus-locked analysis of the N2 in the flanker task.

Given that we did not have a priori hypotheses about the stimulus-locked TT ERPs, analyses were conducted based on visual inspection of the mean amplitudes for each TT condition. This resulted in analyses of the same five central electrodes across four 100 ms time bins: 50–150, 150–250, 250–350, and 350–450 ms. Mean amplitude in these time bins was analyzed within a 5 (Channel Position) × 4 (Time Bin) × 2 (TT Condition) repeated-measures ANOVA.

***Scalp topographies.*** In order to examine differences across conditions and channel locations, scalp topographies were generated in two ways. First, the mean amplitude difference between conditions was depicted across channel locations. Given that such differences sometimes include a summation of multiple generators, a Laplacian transformation (i.e., current source density, CSD) was performed on the data in order to qualitatively assess differences in scalp distributions across conditions. Following Riés et al. (2011), Laplacian transformations were performed on individual subject averages in Brain Vision Analyzer (3° spline, 15° maximum Legendre polynomial), then these individual transformations were averaged and the differences between conditions were computed. Although CSD is not optimal for detecting deep sources such as the ERN, the method has been used previously in studies of the ERN and N2 (e.g., Nieuwenhuis et al., 2003; Riés et al., 2011), and differences that emerge in CSD topographies are suggestive of different generators.

#### EEG – Behavior correlations

In order to assess whether there was a relationship between behavioral and EEG signatures of conflict, correlations were conducted between the flanker and TT effects (i.e., TT – non-TT). Behavioral effects for the flanker task included people’s overall reaction time and accuracy as well as the mean differences in reaction time and accuracy between congruent and incongruent trials. For the TT task, overall error rates and proportions of errors that were self-corrected were included. TT effects (i.e., TT– non-TT) were also included for each of the three types of errors coded, as well as the proportion of errors that were self-corrected.

For EEG signatures of the flanker task, the mean amplitude differences were calculated for the N2 and ERN components as described above. For the TT task, the ERN was calculated in the same way. Differences between TT conditions were generated based the mean amplitudes for TT and non-TT conditions across the time windows where there was a significant TT effect: 50–350 ms (see Results). For all correlation analyses, estimates of differences were done at electrode FCz as this electrode consistently showed differences between conditions.

## RESULTS

### BEHAVIORAL

#### Flanker Task

**FIGURE 1:**
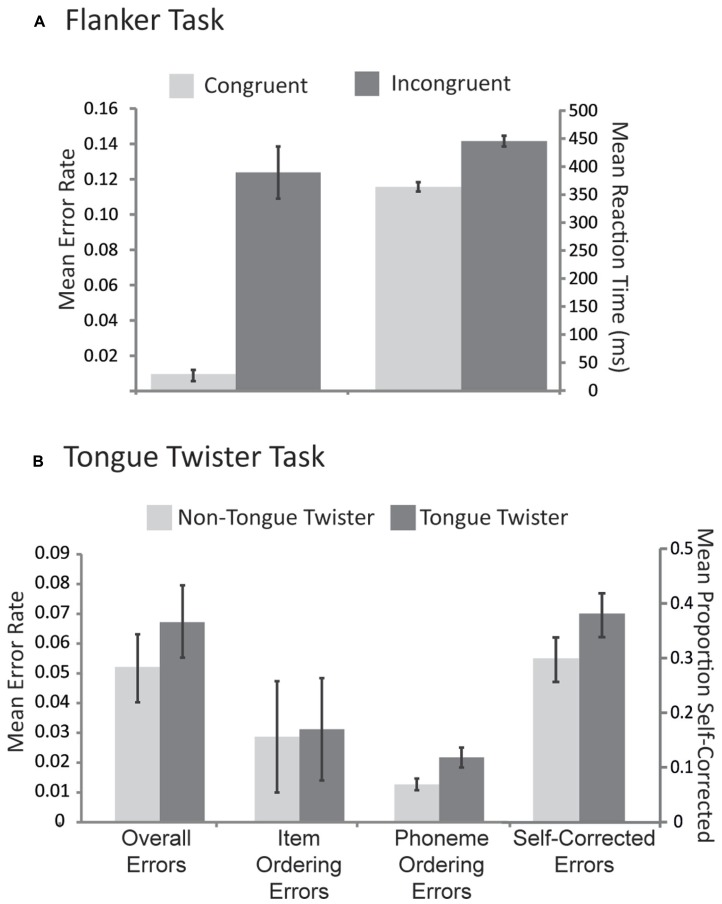
**Behavioral results across both tasks. (A)** Mean error rates and reaction times for congruent and incongruent trials in the flanker task. **(B)** Mean error rates for three different types of errors in the tongue twister task as well as the proportion of errors that were self-corrected across each tongue twister condition. Error bars correspond to standard error across participants.

Mean reaction times and error rates to congruent and incongruent stimuli are provided in **Figure 1A**. The pattern of results replicates previous research as participants were both slower [μ_D_ = 79.5 ms, SD_D_ = 19.5 ms; *t*(43) = 25.2, *p* <0.001] and less accurate [μ_D_ = 11.2%, SD_D_ = 6.6%; *t*(43) = 10.9, *p* <0.0001] for incongruent relative to congruent trials.

#### Tongue Twister Task

Mean error rates for each TT condition across different error types are provided in **Figure 1B**. For the overall proportion of errors, there was an effect of TT condition, with more errors for TT relative to non-TT trials [μ_D_ = 1.1%, SD_D_ = 2.5%; *t*(39) = 2.7, *p* < 0.05]. No such pattern emerged for item ordering errors [μ_D_ = 0.2%, SD_D_ = 1.8%; *t*(39) = 0.39, *p* > 0.8], however, phoneme ordering errors also showed a TT effect [μ_D_ = 0.6%, SD_D_ = 1.8%; *t*(39) = 3.2, *p* <0.01]. In order to examine the efficacy of self-monitoring, we evaluated the proportion of errors that were self-corrected (see **Figure 1B**). Participants self-corrected their errors overall over 30% of the time, and this in turn was influenced by the TT manipulation, with a higher proportion of corrections in the TT than the non-TT condition [μ_D_ = 10.2%, SD_D_ = 27.3%; *t*(39) = 2.14, *p* <0.05]. This result thus indicates that people were better at self-repair in the condition that induced the most errors.

Speakers were instructed to pace their nonword production to produce one nonword per 650 ms. Nevertheless, it is possible that some differences in the onset times between TT and non-TT conditions emerged. Small but reliable differences in speech onset times were observed [μ = 37.1 ms, SD = 20.5, *t*(43) = 11.28, *p* <0.001] such that TT trials were initiated later (mean onset = 448 ms, SD = 36.6) than non-TT trials (mean onset = 411 ms, SD = 40.1). The pattern of errors and response times thus confirmed that TT trials were more difficult than non-TT trials.

**Table 2** contains the distribution of errors across the four nonwords in a sequence, of errors as a function of sequence repetition (**Table 2A**) and the distribution of speech sound errors across syllable positions (**Table 2B**). Examination of **Table 2A** reveals that more errors were likely to occur for the middle two items of a sequence, and error rates increased with increasing repetition. This latter result is consistent with the TT task becoming more difficult with increasing repetition of the items. **Table 2B** contains the distribution of sound-based errors. Here, item-ordering and phonological ordering errors were combined, as the former likely reflects a sound-based error that resulted in production of one of the items. There was an overwhelming tendency for errors to occur in the first syllable position, although the exact location within onset position varied, as did the tendency to self-correct. These results have important implications for the ERN analyses below. The fact that errors were occurring in the first syllable position constrains to some extent the time during syllable production in which an error was happening, suggesting we should observe an ERN. However, the temporal jitter caused by the error occurring at slightly different parts of the onset syllable, in addition to the variation in self-correction may have introduced temporal jitter, leading to a reduction in signal, subsequently leading to a smaller ERN relative to the flanker task. These issues are addressed in the ERP analyses below.

**Table 2:** Distribution of errors across different sequence positions and nonword positions.

(A) Total number of error for each error type in each sequence position.							
	Nonword position with a list				Sequence repetition		
Error type	1	2	3	4	1	2	4
Overall errors	252	316	368	303	164	475	600
Item ordering errors	67	97	91	79	18	132	184
Phoneme ordering errors	109	148	160	103	82	213	225
Self corrections	80	111	125	83	65	172	162
(B) Total number of phonological errors^*^ across tongue twister conditions for each syllable position in a nonword.							
	Syllable position of errors within a nonword		
	Onset	Vowel	Offset				
Tongue twister	499	18	33				
Non-tongue twister	319	S	25				

* Includes both item ordering and phoneme ordering errors.

### EVENT-RELATED POTENTIALS

#### Flanker Task

**FIGURE 2:**
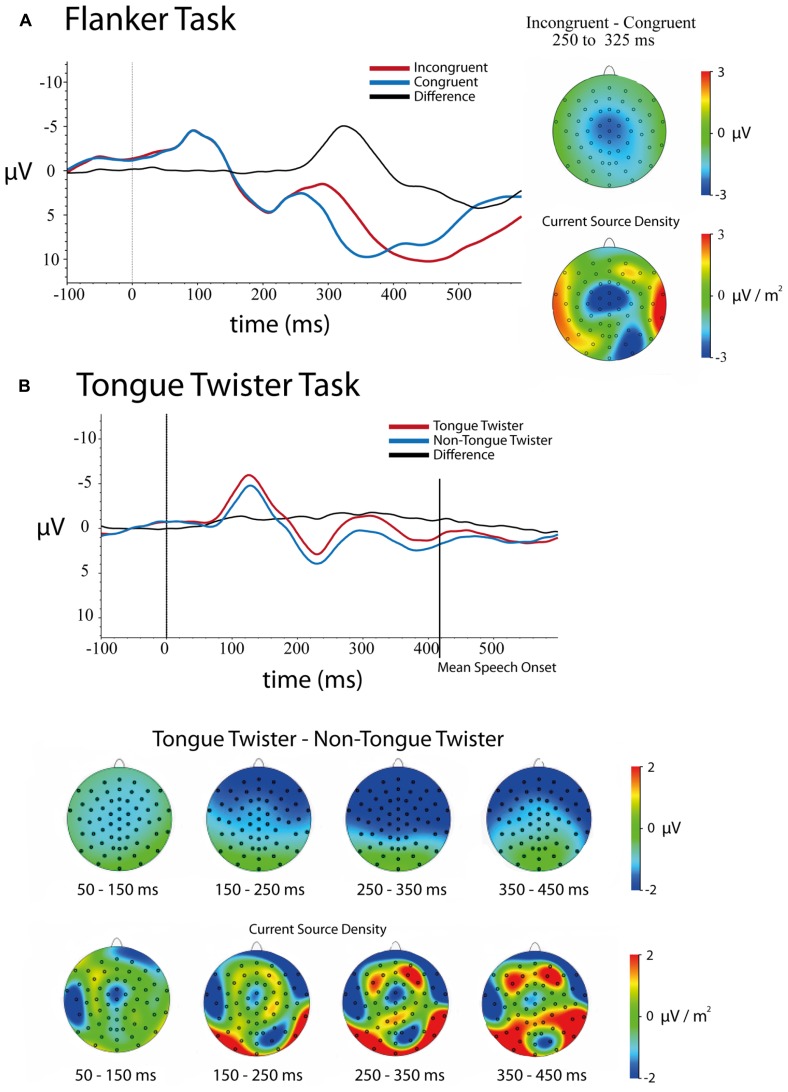
**Stimulus-locked ERPs and scalp topographies of differences for both tasks.** Trials were baseline-corrected -100 ms before stimulus onset. **(A)** Flanker task – congruent and incongruent trials ERPs at electrode FCz as well as scalp topographies of the mean amplitude (upper)and current source density (lower) differences between 250 and 325 ms. **(B)** Tongue-twister and non-tongue twister trials as well as scalp of the mean amplitude (upper) and current source density (lower) differences in 100 ms windows between 50 and 450 ms.

***Stimulus-locked.* Figure 2A** shows the stimulus-locked ERPs at electrode FCz for each of the congruent and incongruent flanker conditions. Results of the 5 (Channel) × 2 (Congruency) ANOVA on mean amplitudes in the 250–350 ms time window following stimulus onset revealed a main effect effect of Channel Location [*F*(4,172) = 83.54, *p* <0.001], main effect of Congruency [*F*(1,43) = 83.39, *p* <0.001], and a Channel × Congruency interaction [*F*(4,172) = 9.61, *p* <0.001]. In line with previous studies of the flanker task, incongruent trials had a larger negative amplitude (μ = 4.78, SD = 0.68) relative to congruent trials (μ = 7.63, SD = 0.69). The main effect of channel was explained by the fact that the frontal channels had a more negative amplitude differences on average than posterior electrodes.

Follow-up analyses at each of the channel locations replicated previous research with a frontal–central distribution to the N2 effect (incongruent – congruent) that was largest over electrodes FCz and Cz, although the effect was significant over all central electrodes tested. The effect at each electrode was as follows: Fz [μ_D_ = -2.35, SD = 2.14, *t*(43) = -7.28, *p* <0.001]; FCz [μ_D_ = -3.16, SD = 2.53, *t*(43) = -8.29, *p* <0.001]; Cz [μ_D_ = -3.12, SD = 2.36, *t*(43) = -8.77, *p* <0.001]; CPz [μ_D_ = -2.97, SD = 2.02, *t*(43) = -9.75, *p* <0.001]; Pz [μ_D_ = -2.68, SD = 1.74, *t*(43) = -10.22, *p* <0.001].

Examination of the scalp topographies largely confirm the frontal–central distribution of this N2 effect, although it is clear in looking at the CSD that there is a frontal and occipital-parietal component in the time window. The latter likely reflect spillover into a difference between conditions into the P3 (see Folstein and van Petten, 2008).

**FIGURE 3:**
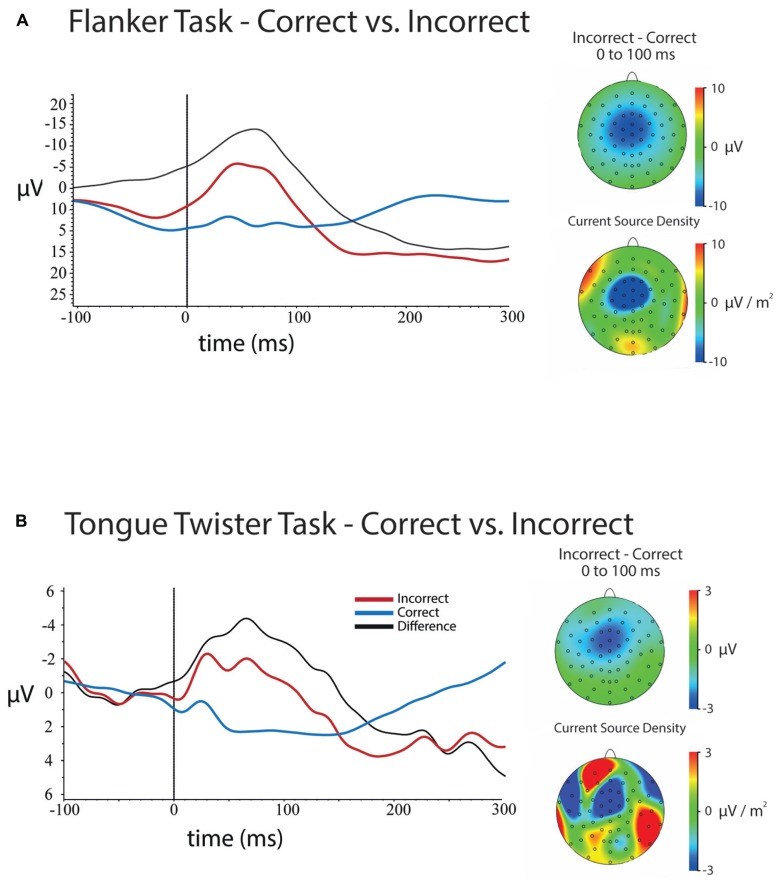
**Response-locked ERPs and scalp topographies of differences for both tasks.** ERPs are plotted for electrode FCz, and were baseline-corrected -300 to -200 ms before response onset. **(A)** ERPs for errors and correct trials at FCz in the flanker task and the scalp topographies of the mean amplitude (upper) and current source density (lower) differences between errors and correct trials between 0 and 100 ms after a response. **(B)** ERPs for errors and correct trials for the tongue twister task at FCz as well as scalp topographies of the mean amplitude (upper) and current source density (lower) differences between errors and correct trials between 0 and 100 ms after a response.

***Response-locked.*** **Figure 3A** shows the response-locked ERPs for errors and correct trials for the flanker task at electrode FCz. Results of the 5 (Channel) × 2 (Error) ANOVA on mean amplitudes for the 100 ms following response onset revealed a main effect of Channel Location [*F*(4,172) = 118, *p* <0.001], a main of effect of Error [*F*(1,43) = 106, *p* <0.001] and a Channel × Error interaction [*F*(4,172) = 33.58, *p* < 0.001].

The main effect of Errors came from the fact that the mean amplitude was more negative for errors (μ = 2.59, SD = 0.88) compared to correct trials (μ = 10.74, SD = 0.76). The main effect of channel location came from the fact that the mean amplitudes over frontal electrodes were more negative than posterior electrodes.

Examination of the scalp topographies and follow-up analyses at each of the channel locations showed that the ERN (error – correct) had a frontal–central scalp distribution that was maximal over electrode FCz, although significant over all central electrodes that were tested. The effect at each electrode was as follows: Fz [μ_D_ = -7.99, SD = 6.03, *t*(43) = -10.54, *p* < 0.001]; FCz [μ_D_ = -10.33, SD = 6.17, *t*(43) = -11.11, *p* < 0.001]; Cz [μ_D_ = -9.43, SD = 6.01, *t*(43) = -10.40, *p* < 0.001]; CPz [μ_D_ = -7.35, SD = 5.53, *t*(43) = -8.81, *p* < 0.001]; Pz [μ_D_ = -5.64, SD = 4.97, *t*(43) = -7.52, *p* < 0.001].

#### Tongue Twister Task

***Stimulus-locked.*** **Figure 2B** contains the ERPs over electrode FCz as well as the scalp distribution of differences for TT and non-TT conditions. A 5 (Channel Location) × 4 (Time Bin) × 2(TT Condition) repeated-measures ANOVA on mean amplitudes revealed main effects of Time [*F*(3,117) = 28.40, *p* < 0.001], Channel [*F*(4,156) = 13.05, *p* < 0.001] and TT Condition [*F*(1,39) = 7.19, *p* < 0.02] as well as interactions between Time × Channel [*F*(12,468) = 5.72, *p* < 0.001], Channel × TT Condition [*F*(4,156) = 7.49, *p* < 0.001] and Time × Channel × TT Condition [*F*(12,468) = 3.54, *p* < 0.001]. The only interaction that was not significant is Time × TT Condition [*F*(3,117) = 1.026, *p* > 0.3].

The main effect of Time simply indicates that the average amplitude varied across the four time bins (See **Figure 2B**). The main effect of Channel came from the fact that there was a gradient in mean activity from negative to positive going from anterior to posterior channels (mean amplitude: Fz = -0.62, FCz = -0.15, Cz = -0.14, FPz = 0.25, Pz = 0.76). As can be seen in **Figure 2B**, the main effect of TT Condition came from the overall more negative amplitude for TT trials (μ = -0.48, SD = 0.52) relative to non-TT trials (μ = 0.52, SD = 0.46).

As can be seen in **Figure 2B**, the Channel × TT interaction came from the fact that the difference between TT conditions was larger over frontal compared to posterior electrodes. Mean differences between TT and non-TT conditions at each channel were as follows: Fz [μ_D_ = -1.26, SD = 0.55]; FCz [μ_D_ = -1.21, SD = 0.51]; Cz [μ_D_ = -1.04, SD = 0.51]; CPz [μ_D_ = -0.85, SD = 0.48]; Pz [μ_D_ = -0.65, SD = 0.47]. The Time × Channel interaction was not of theoretical importance, and simply indicates that the relative difference in mean amplitude across channels varied over time.

In order to breakdown the three-way interaction between Channel × Time × TT, separate repeated measures ANOVAs were run between the Channel and TT factors at each time bin. Results of these ANOVAs showed that no Channel × TT interaction was observed in the time windows 50–150, 150–250, and 350–450 ms. Thus, the only time window showing an interaction between Channel × TT was 250–350 ms [*F*(4,156) = 7.69, *p* < 0.001]. As with the overall interaction between these two factors, the TT effect was largest over frontal electrodes compared to posterior electrodes. Finally, it should be noted that a TT effect was observed in every time window except the final time window (350–450 ms), which includes the average speech onset for both conditions [*F*(1,39) = 2.07, *p* > 0.15]. Thus, the difference between TT conditions lasted up until speech onset.

Looking across these results, it is clear that there were very early effects of the TT manipulation that lasted throughout the speech planning process up to the point of articulation. The difference in mean amplitude generally had a frontal scalp distribution, although examination of the current source densities reveals that this overall scalp distribution is likely the result of many generators summing together. This pattern makes it difficult to assess the stages of production planning at which these differences are arising. What is clear, however, is that the patterns observed across correct TT trials do not parallel the sort of clear N2 difference that was observed between congruent and incongruent flanker trials. We return to this in the general discussion.

***Response-locked.*** **Figure 3B** contains ERPs and the scalp topography of the difference between errors and correct trials at electrode FCz. Results of the 5 (Channel) × 2 (Error) ANOVA on the mean amplitude difference between correct and error trials for the 100 ms following vocal onset revealed a main of effect of Error [*F*(1,29) = 11.03, *p* < 0.005], a Channel × Error interaction [*F*(4,116) = 8.46, *p* < 0.001], but no main effect of Channel Location [*F*(4,116) = 1.62, *p* > 0.3].

As expected, an ERN was observed, and error trials significantly more negative overall (μ = -0.50, SD = 0.76) than correct trials (μ = 1.52, SD = 0.46). Examination of **Figure 3B** and follow-up analyses showed that the difference in amplitudes between error and correct trials had a frontal central scalp. This difference was maximal over electrode FCz, although differences were significant all electrodes except Pz. Mean differences between error and correct trials were as follows: Fz [μ_D_ = -2.36, SD = 3.88, *t*(29) = -3.33, *p* <0.005]; FCz [μ_D_ = -2.91, SD = 4.27, *t*(35) = -3.74, *p* <0.001]; Cz [μ_D_ = -2.28, SD = 3.53, *t*(35) = -3.55, *p* <0.001]; CPz [μ_D_ = -1.61, SD = 3.04, *t*(35) = -2.90, *p* <0.01]; Pz [μ_D_ = -0.97, SD = 2.83, *t*(35) = -1.87, *p* <0.07].

In examining **Figure 3B** it is clear that although an ERN was observed, the mean amplitude was much smaller was more temporally spread than in the flanker task. Furthermore, although the scalp topography showed that the ERN was centered over FCz as is typically observed for this component, the CSD reveals that there are likely multiple frontal components that are combining to generate the average difference. The smaller amplitude and temporal profile is likely a result of two factors. First, there were simply fewer trials on average for the TT ERN relative to the flanker ERN. Second, as noted above, although the majority of errors occurred at syllable onset position, there was still substantial temporal variation in the onset of errors. These time differences likely introduced some noise into the estimate of the ERN amplitude, and may have implications for the correlations reported below.

### ERP-BEHAVIOR CORRELATIONS

**Table 3** contain the correlations between behavioral performance on both the TT and flanker task with ERP markers of errors in both tasks (i.e., the ERN), the N2 difference in the flanker task (incongruent – congruent), as well as stimulus-locked differences between TT and non-TT trials. Note that the pattern of correlations identified as “significant” would not survive correction for multiple comparison. Nonetheless, we include these correlations here as they provide a window into the domain-generality of signals of conflict and errors. What is noteworthy about the pattern in the table is that there are very few significant correlations overall, and virtually nothing significant between the two tasks.

**Table 3:** ERP-Behavior correlations across flanker and tongue twister tasks^*^^+^.

		Tongue twister effect (TT non-TT)				Tongue twister overal				Tongue twister ERP		Flanker ERP	
		Error proportion	Item ordering errors	Phoneme ordering errors	Self corrections	Overall error proportion	Item ordering errors	Phoneme ordering errors	Self corrections	TT non-TT stimulus locked	TT ERN	N2	ERN
**Flanker ERP**	**N2**	-0.03	-0.10	0.01	0.04	0.10	-0.11	0.10	-0.15	0.15	0.04		**0.42**
	ERN	-**0.29**	-*0.25*	-0.08	0.12	-0.05	0.00	-**0.25**	-*0.22*	-0.02	-0.17		
Flanker behavior	Congruency RT	-0.11	-0.12	-0.11	**0.44**	0.12	-0.13	0.19	0.12	0.01	0.04		
	Congruency Error Rate	0.11	**0.30**	-0.19	-0.02	0.13	-0.02	0.22	0.07	0.04	0.25	0.16	-0.04
	Average RT	0.06	0.00	0.05	0.04	0.18	0.14	0.03	-0.12	-0.12	0.03	0.13	0.01
	Average Error Rate	0.12	*0.29*	-0.18	-0.02	0.12	0.00	0.19	0.06	0.04	0.25	0.07	-0.02
TT ERP	TT non-TT Stimulus Locked	-0.27	-0.09	-0.32	-0.22	0.10	0.08	-0.01	-0.25				
	ERN	*0.31*	**0.37**	0.24	0.01	0.23	0.18	0.23	0.12	-0.07			

* Bold font indicates correlations with *p* <0.05; Italic indicates *p* <0.10 (all effects uncorrected for multiple comparisons)

+ *N* = 44 for Flanker effects; *N* = 40 for tongue twister effects; *N* = 30 for the tongue twister ERN.

There are a few patterns, however, that are worth mentioning. First, the correlation between the N2 and ERN in the flanker task replicates previous findings (e.g., Yeung et al., 2004). Second, the ERN in the TT task was correlated with the TT effect (TT-non-TT) for item ordering errors, and was marginally significant for the TT effect for errors overall. Third, there were negative correlation between the ERN in the flanker task and the TT effect (TT-non-TT) for the overall error proportions, as well as marginally significant correlations with the TT effect for item ordering errors and the overall number of phoneme ordering errors. Fourth, there were two instances of significant, cross-task correlations: a positive correlation between the reaction times for the congruency effect in the flanker task and the TT effect in the likelihood that people self-corrected their errors, and a positive correlation between the congruency effect for error rates and the TT effect for item ordering errors. Finally, and importantly, there was no correlation between the ERNs in the flanker and TT task. This lack of correlation could genuinely reflect differences between tasks, or may be partly a result of the noise introduced in the ERN for the TT task as a result of temporal jitter in the onset of speech errors. We return to this point as well as the overall lack of correlation across tasks in the general discussion section that follows.

## GENERAL DISCUSSION

The present study was designed to test whether monitoring in language production might be achieved through a domain-general, response conflict monitoring system. EEG was recorded while people performed the TT and flanker tasks, linguistic and non-linguistic tasks designed to elicit varying degrees of response conflict. In order to test whether similar mechanisms were present in both tasks, EEG and behavioral signatures of response conflict and error commission were quantified and compared both qualitatively and quantitatively. Results showed that both the flanker and TT tasks elicited a centrally-distributed ERN. Replicating previous results, incongruent trials in the flanker task elicited a larger N2 than congruent trials. Critically, no such signature was present in the TT task, in which a broadly distributed frontal negativity was observed for the difference between TT and non-TT conditions. ERP-behavior correlations showed some correlations within a task, but few between the two tasks. Taken together, although these results do not necessarily speak against conflict-monitoring as a mechanism for monitoring in speech production, they do not support a common-locus to this mechanism across task domains. These results have implications for the breadth of the conflict-monitoring hypothesis generally, and monitoring within language-production more specifically.

The current study replicates and extends previous research in a number of ways. With regards to the flanker task, we replicated the N2 difference for congruent and incongruent trials, the ERN for correct and incorrect trials, and importantly, the correlation between these two EEG signatures. This latter result is consistent with the conflict monitoring hypothesis (see Yeung et al., 2004), but also a more recent account that attributes the N2 and ERN signals to an action-outcome prediction process (Alexander and Brown, 2011), a theoretical point we return to below.

Although the TT task has not previously been studied with EEG, the current pattern of results also bears similarity to previous studies using other error elicitation paradigms. Similar to the Masaki et al. (2001) study using a vocal version of the Stroop task, we also observed an ERN for errors compared to correct trials. The timing and maximal scalp topography of the ERN in the present investigation is very similar to that which was observed in by Masaki et al. (2001) and is somewhat broader than the ERN observed for the flanker task. Importantly, the ERN in the current study was positively correlated with the TT effect for item ordering errors, and marginally with people’s overall error rates, confirming that there is a relationship between the generator of the ERN and people’s self-monitoring abilities. The combination of these results suggests that the ERN in language production may reflect the activity of a continuous performance monitoring system.

In addition to the ERN, there was a sustained difference between TT and non-TT trials prior to responding. This sustained difference parallels that which has previously been observed preceding error trials in the SLIPs paradigm (Moeller et al., 2007; Severens et al., 2011). In contrast to the studies of the SLIPs paradigm, the time course of the differences began earlier (50 ms) and were sustained up until just before speech initiation began. This difference likely reflects the summation of multiple potentials, as evidenced by the scalp distribution of the CSD. 

The timecourse and distributed nature of TT effect make it difficult to pinpoint when differences between TT conditions were occurring during production planning. Despite the TT conditions being matched across participants (i.e., different participants saw the same items in tongue-twister and non-TT versions), it remains possible that some of the differences reflect differences in reading the pseudowords. Although it is beyond the scope of the current study to fully address this issue, it is possible that some of the very early effects reflect differential sensitivity in the N170 to visual properties during reading, and later differences with accessing the phonological properties of words in the N320 time window (see Bentin et al., 1999 for a review). Regardless of the specific source (i.e., in reading or production planning), these results suggest that the difference between the two TT conditions are unlikely to be driven solely by the same generators that are implicated in the flanker task. In turn, this suggests that the phonological nature of the conflict in the TT task that was occurring prior to responding may reflect a form of representational conflict instead of response conflict. We return to this discussion below, but first address one of the central issues for which this study was designed: whether monitoring in production is accomplished via a domain-general, response conflict monitor.

One of the central predictions of conflict-monitoring hypothesis is that a region of the medial prefrontal cortex, the ACC, serves as a domain-general conflict monitor (Botvinick et al., 2001). The domain-generality of the ACC functionality has been adopted by recent models of monitoring in language production (Nozari et al., 2011), and also has been emphasized in a number of papers studying language production as well (e.g., Rodriguez-Fornells et al., 2006; Hirschfeld et al., 2008; Riés et al., 2011; Acheson et al., 2012). The relationship of the ACC to the electrophysiological markers used in this study (the N2 and ERN) has been well-established outside of language (see Yeung et al., 2004), thus the current research provides an important test of this domain-generality. Previous research that has implicated similar electrophysiological markers of ACC response have used testing conditions that either used the same task with different motor effectors (e.g., Holroyd et al., 1998), or different tasks with the same motor effectors (e.g., Riesel et al., 2013). Similar to these previous results, there was a correlation between the N2 and ERN in the flanker task. Importantly, however, the ERNs were not correlated across tasks.

There are, of course, a number of reasons why we may have failed to find significant correlations. For instance, the ERN generated in the production task was likely jittered in time compared to the flanker task, given that errors occurred at multiple points in a nonword. Another possibility is that the signal-to-noise ratio was worse in the TT task as the ERN was calculated from fewer error trials. A third possibility is that the lack of correlation is a result of different response modalities (i.e., verbal vs. manual; but see Holroyd et al., 1998). These less interesting points aside, one intriguing possibility is that people may be differentially sensitive to different types of errors, which would suggest some amount of domain-specificity to the error monitoring process. Of particular note here is the Laplacian transformation (i.e., CSD), which showed a standard, frontal–central ERN in the flanker task, but more distributed frontal and central generators of the ERN in the TT task. While this suggests some differences between the two generators, CSD is not an optimal measure for finding deep sources such as the ACC, and the current study was not optimized to directly test for differences in source location. If the observed differences are genuine, however, the current results have important implications for theories of action monitoring as the ERN has been taken as a biomarker of error-processing outside of language. Not only does the lack of correlation in the ERN across manual and vocal responding fail to support a domain-general conflict monitor, it is also not consistent with a single, domain-general, error-monitoring system (e.g., Holroyd and Coles, 2002). While this result is potentially interesting, it is a null result, and will need to be tested in future experimentation using a more spatially-sensitive measure (e.g., fMRI), and possibly in tasks that utilize the same output modality.

The present investigation adds to a growing body of evidence that has failed to find evidence of a domain-general, conflict monitoring system. Research using standard cognitive control tasks, for instance, have failed to find that correlations in people’s behavioral response to conflict across a range of conflict-inducing tasks (e.g., Stroop; Fan et al., 2003; Keye et al., 2009). Furthermore, attempts to find cross-task, conflict-adaptation effects have often failed to find any spillover effects from one task onto another (see Egner, 2007), as have those that have tried to find cross-task interference by virtue of factorial designs between two conflict-inducing tasks (e.g., a combined Simon-Flanker task; see Egner, 2008). Finally, attempts to relate the neural signatures of responses of conflict have revealed that while there may be some spatial overlap in the brain response to conflict across tasks within the ACC, there are also noteworthy differences in the spatial location of these responses (Fan et al., 2003).Taken together, these results have led researchers to conclude that while the mechanisms of conflict monitoring might be domain-general, the actual brain regions implementing this monitoring and exerting control may be domain-specific (Milham et al., 2001; Egner, 2008). An alternative view could be accommodated within Alexander and Brown’s (2011) PRO model, which equates ACC activation to a process of comparing predictions about likely action outcomes to the action that is taken. Here, one could assume that this process of prediction and comparison is domain-general, but that the actual action domains over which possible outcomes are being predicted are domain specific.

Such a view would be broadly consistent with neuroimaging studies that have implicated different regions of the ACC for conflict- and error-monitoring (Garavan et al., 2003), as well as studies that have demonstrated different functional subdivisions within the ACC depending on task and motor output (Paus et al., 1998). Critically, the activity of the ACC seems to be related to situations in which there is response- but not representational conflict (Milham et al., 2001; but see Aarts et al., 2008). Within linguistic representation and responses, for instance, a study using a lexical-decision task with bilinguals found that lateral prefrontal regions were sensitive to stimulus-based (i.e., representational) conflict, while the ACC became active only when this conflict led to different responses (van Heuven et al., 2008). Representational conflict here is a form of conflict that would arise pre-response, for instance, as a result of lexical or semantic interference between color words and ink color in the Stroop task. Such pre-response, representational conflict may be functionally equivalent to the competition that is at the heart of selection mechanisms in models of language production (e.g., Levelt et al., 1999). Numerous studies have implicated that other frontal brain regions, such as the inferior frontal cortex (IFG) are sensitive to representational conflict arising due to incompatible stimulus representations (e.g., Stroop interference) or situations of under-determined responding (e.g., in verb generation; see Novick et al., 2005). With regards to monitoring and control operations more generally, these studies point to a network of brain regions involved in the monitoring, detection and response to conflicting stimulus and response dimensions. These networks, in turn, may be organized as separable systems depending on the representational source (e.g., visual, verbal) and the motor effectors (e.g., manual, articulatory) influenced by conflict. The tasks used in the current study maximally differentiated both representational and response conflict, thus future experimentation might be focused on manipulating only one of these two sources (e.g., using verbal responding across different sources of representational conflict), or through conflict adaptation paradigms, such as task switching or factorial manipulations of similar sources of conflict.

Bringing this back to a conflict-monitoring hypothesis for language production, the model put forward by Nozari et al. (2011) suggested that conflict signals can be generated both at the level of lexical-retrieval and phonological encoding by, for instance, calculating the difference between the two most active elements at the time of selection. Although the authors discussed this research within a larger, response conflict framework, the present results suggest that it may be more appropriate to discuss these signals in terms of pre-response (i.e., representational) conflict. This does not diminish the fact that signals within the production system itself might serve as cues to monitoring and the need to increase cognitive control. Rather, there may be more domain-specificity to this signal than was originally emphasized. The current study thus suggests that it is unlikely that all tasks domains are mapping onto the exact same conflict-monitor. What remains possible, however, is that the signal to increase monitoring and control could be domain-general, but that it would originate from domain-specific systems and stages of planning. Put differently, the current research suggests a model of monitoring in speech production in which signals arising at many different levels of the planning process (e.g., message, grammatical, lexical, phonological, articulatory) could serve as cues to increase monitoring and control. Each of these signals would, presumably, originate in the cortical networks responsible for each of these stages, and the signals themselves might be mediated via distinct temporal, medial frontal, lateral frontal, and midbrain loops (see Alexander et al., 1986 for a review of cortical-midbrain loops). Ultimately, the extent to which this might lead to response conflict in particular is dependent upon whether activation of two competing response alternatives cascade all the way to articulatory planning.

Although the current paper and discussion has been motivated by a response conflict monitoring perspective, the present results might also be accommodated within a recent account suggesting that signals for monitoring and increased cognitive control could emerge as a result of prediction errors (see Alexander and Brown, 2011). This latter account differs from a conflict monitoring hypothesis in a number of ways, not the least of which is that it requires some mechanism of comparing intended with actual responding. According to the PRO model, the signal that arises from incongruous conditions does not arise from conflict, *per se*, but rather, because multiple responses have been predicted, and only one of them was executed. The difference between predicted and actual responding results in a prediction error. This proposed mechanism is, in many ways, similar to recent proposals by Pickering and Garrod (2013), who suggest that prediction serves a central role in production planning and monitoring. The mechanism of prediction in the PRO model is the learned association between a stimulus to possible response outcomes, and not from response to possible sensory consequence. As such, prediction in the PRO model bears more similarity to what Pickering and Garrod (2013) refer to as prediction by association rather than prediction via forward models.

Framing this prediction-based account within the staged process of language production, “response” in this instance could be viewed as what is sent from one planning stage to another. Given that this process is likely to be cascaded, the prediction-error account suggests that partially processed information from one stage of production planning (e.g., message-retrieval) would lead to “predictions” (i.e., partial activation) of items at the next stage of planning (e.g., multiple lexical candidates). Ultimately, one of these candidates is selected, thus leading to a form of prediction error at the following stage of production planning. Whether the above-described comparison process necessarily requires the comprehension system (as proposed in the Perceptual Loop theory; Levelt, 1983) remains to be determined. It does suggest, however, that there should be a high degree of domain-specificity with regards to the predictions that are being made. In the current study, for instance, the use of nonword stimuli emphasized phonological encoding over lexical-semantic representation, which was done to elicit conflict primarily at late stages of the production planning process. Such monitoring may or may not be different from that which is involved in monitoring at the lexical-semantic level, and this remains an important area for future investigation.

To conclude, the current study sought to test whether a domain-general, conflict monitoring mechanism might serve as a cue to monitoring in speech production. By comparing EEG and behavioral signatures of conflict across both speech and non-speech tasks, the present results provide little evidence in favor of a single, conflict monitoring mechanism. Similar findings have been observed outside of language. This pattern of results thus suggest that while the mechanism and signals to increase monitoring and control may be domain general, the representations over which they are generated are likely to be domain-specific.

## Conflict of Interest Statement

The authors declare that the research was conducted in the absence of any commercial or financial relationships that could be construed as a potential conflict of interest.
